# Modification of the Railway Traction Elements

**DOI:** 10.3390/ma16082941

**Published:** 2023-04-07

**Authors:** Jarosław Konieczny, Krzysztof Labisz, Wojciech Pakieła

**Affiliations:** 1 Department of Railway Transport, Faculty of Transport and Aviation Engineering, Silesian University of Technology, 44-100 Gliwice, Poland; 2 Department of Materials Engineering and Biomaterials, Faculty of Mechanical Engineering, Silesian University of Technology, 44-100 Gliwice, Poland

**Keywords:** section insulator guide, Cu-ETP, laser alloying, wear treatment, hardness, X-ray, SEM, TEM

## Abstract

This paper presents the results of research on a newly developed surface layer made by laser remelting the working surface of the Cu-ETP (CW004A, Electrolytic Tough Pitch) copper section insulator guide with Cr-Al powder. For the investigation, a fibre laser was used with relatively high power, reaching 4 kW, so as to ensure a high gradient of cooling rate for microstructure refinement. The microstructure of the transverse fracture of the layer (SEM) and the distribution of elements in the microareas (EDS) were investigated. The test results showed that chromium does not dissolve in the Cu matrix, and its precipitates take the shape of dendrites. The hardness and thickness of the surface layers as well as the friction coefficient and the influence of the Cr-Al powder feeding speed on them were examined. For the distance from the surface to 0.45 mm, the hardness of the produced coatings is above 100 HV_0.3_, while the friction coefficient of the produced coatings is in the range of 0.6–0.95. More sophisticated investigation results concern the d-spacing lattice parameters of the crystallographic structure of the obtained Cu phase reaching the range between 3.613–3.624 Å.

## 1. Introduction

Copper is highly reflective to infrared-wavelength light, which is the typical wavelength of almost all the laser sources mounted on MAM machines today (i.e., ~1080 μm). As a result, Cu powder is not able to absorb the energy required for melting, so to overcome this critical issue, more energy should be supplied by using more powerful laser sources. For this reason, the undertaken research was carried out to minimize surface properties [1].

Section insulator guides play an important role in railway transportation; they are especially of huge importance when the fluency and stability of an electric current in the grid should remain stable. They should be built of material that presents excellent electrical conductivity and their surface should ensure optimal mechanical properties such as hardness, wear resistance, and corrosion resistance in elevated temperature because of the occurrence of an electric arc. Some parameters can still be improved, especially the plastic deformation stability, which is the main reason for exchanging used and deformed parts.

The section insulator guides and the wires of the railway traction are made of copper (CW004A) Cu-ETP [2,3]. During operation, they are exposed to several tribological mechanisms, including corrosion (oxidation of copper), friction, abrasive wear, and arc erosion [4], the most destructive of which is an electric arc [5]. The generation of an electric arc causes disturbances in current flow, a rapid increase in temperature at the contact points [6], which leads to increased wear of the guides and the phenomenon of micro-welding [7]. Changing the geometry of the guide and changing the smoothness of the surface contribute to the more frequent occurrence of an electric arc [8].

Therefore, it seems advisable to strengthen the working surface of the guide that is in contact with the pantograph current collector of the pantograph. The result of such a modification of the surface should be an increase in tribological resistance and resistance to high temperature induced within the electric arc [9]. Modification of the guide surface should also reduce the electrical conductivity of the guide as little as possible.

One of the methods used to modify the structure and properties of the surface layers is alloying by using a laser beam [10]. The first attempt to use a laser for the enhancement of the surface layer microstructure was done using a CO_2_ laser. Due to some disadvantages and also the availability of new laser technology in this work, a fibre laser was used. Fibre lasers have advantages concerning good output laser beam quality, as well as high electro-optic efficiency, a wide range of work materials, low comprehensive operating cost, and showcase higher technical and economic performances. Compared with CO_2_ lasers, fibre lasers have a higher conversion efficiency and lower use cost. According to the economic calculation, the use cost of fibre lasers is 50% lower than the usage cost of CO_2_ lasers per hour. Fibre lasers have higher power, efficiency, and also do not require any adjustment or maintenance.

Fabrication of metallic matrix composite (MMC) coatings by adding ceramic powders often suffer from weak interfacial bonding due to poor wetting, formation of undesirable interfacial products, and nonuniform distribution of the ceramic phase in the metallic matrix. In order to solve such a problem, the composite coatings are fabricated in-situ [11].

As a result of the laser introduction of Cr-Al particles into the surface layer of the guides of sectional insulators made of Cu-ETP copper, the structure of the surface layer changes, and the properties are improved while maintaining thermal and electrical performance to those of existing and functioning insulators.

Since Cu-ETP melts at a temperature about 800 °C lower than the Cr melting point [4], the optimal selection of the process parameters is crucial to achieve complete sludge melting and obtain the desired microstructure and composition in AZ. Additionally, both elements Cu and Cr differ in values in terms of thermal conductivity, specific heat, or density [4,12].

The aim of this publication is to investigate the functional properties of the guide layer made of Cu-ETP copper alloyed with CrAl powder by means of a high-power fibre laser beam.

## 2. Material and Investigation Methodology

### 2.1. Laser Treatment Parameters

**Figure 1:**
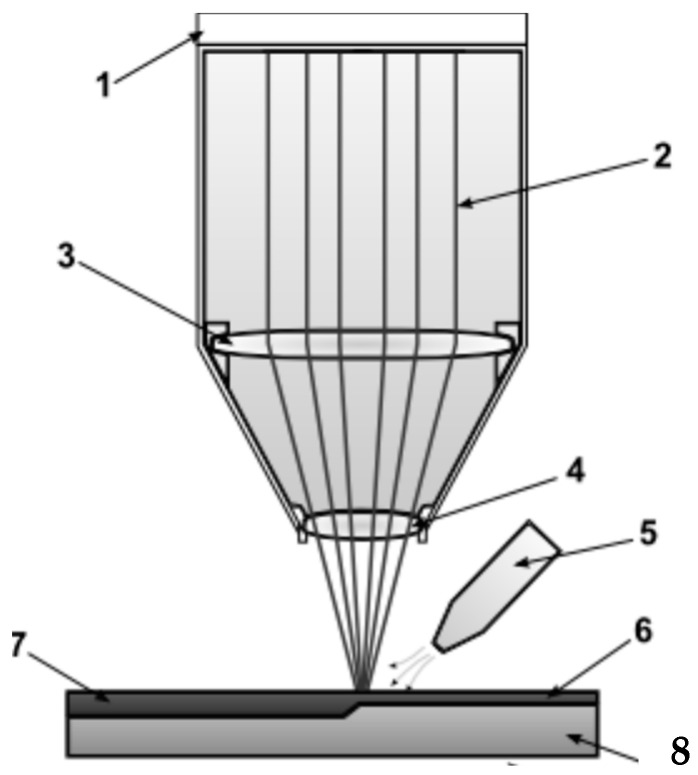
Schematic diagram of the stand for laser feeding of metal powder to the top layer of the insulator guide: 1—laser head, 2—laser beam, 3—focussing lens, 4—protective glass, 5—protective gas nozzle, 6—applied layer alloying material, 7—alloyed layer, 8—native material.

**Figure 2:**
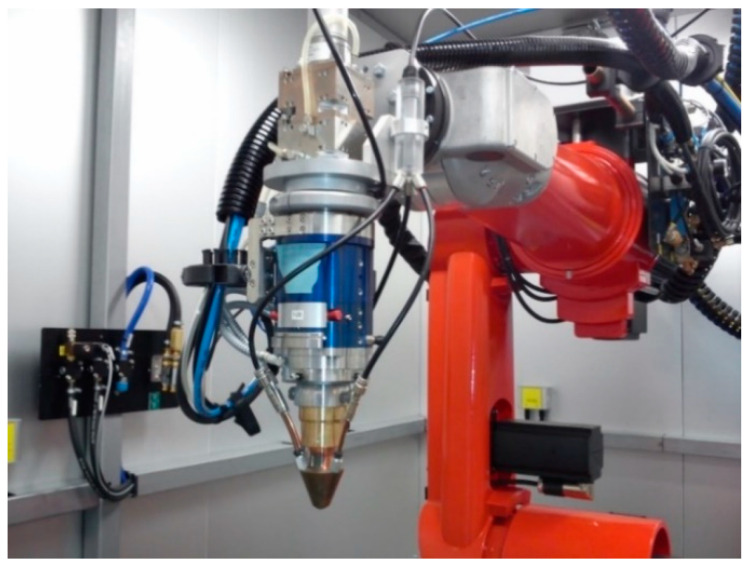
Applied fibre laser.

Laser remelting was performed by feeding the powder with a speed in the range of 0.2–0.8 mm^3^/min in a continuous manner to the area of the molten metal pool by dosing the granulate using a fluidized feeder. The powder feeder was connected to the transport gas cylinder and the powder feed nozzle. The feathering was made in an argon shield to protect the substrate against oxidation (Figure 1). A fibre laser (GOPhotonics, New Delhi, India) was used to remelt the working layer of the guide (Figure 2). CrAl powder in a ratio 95% Cr to 5% Al was used as the alloying material.

Based on the macrostructure analysis of the remelted surface layer, the optimal laser power P = 4 kW for the Cu-ETP copper substrate material and the appropriate speed of fusion and remelting the guide were determined. It was found that the optimal speed of the laser beam V_skan_ = 0.05 m/min. The powder feed rate was 0.2 mm^3^/min, 0.4 mm^3^/min, and 0.8 mm^3^/min.

### 2.2. Microstructure Analysis

The grain microstructure and disorientation studies were performed on the ZEISS SUPRA 25 scanning electron microscope (SEM—Zeiss, Oberkochen, Germany) using the EDS and EBSD methods. Samples for SEM observation in EBSD mode were prepared on a GATAN ion polisher. For phase identification, the Titan 800 transmission electron microscope (FEI, Eindhoven, The Netherlands) was used with the field electron gun X-FEG with a brightness reaching 5·10^7^ A·m^−2^·sr^−1^ V^−1^ and electron energy ranging from 80 to 300 kV, especially using the selected area diffraction patterns (SAED).

### 2.3. Hardness Measurement

Hardness was measured on a Zwick/ZHR hardness tester (ZwickRoell, Ulm, Germany) using the Rockwell method. The indenter load was 590 N, and the indenter diameter was 1/16 inch. The hardness tester is equipped with an electronic module to calculate the HV hardness.

### 2.4. Wear Treatment Tests

The abrasion resistance test was performed on a Taylor Hobson tribometer (Taylor Hobson Ltd., Leicester, UK). The ball-shaped counter sample with a diameter of 6 mm was made of ZrO_2_ and the load was 10 N. Each measurement consisted of 1000 cycles across the path length of 7.2 mm. The total distance that the sample made each time was 36 m.

## 3. Investigation Results

### 3.1. Scanning Microscope Investigation Results

**Figure 3:**
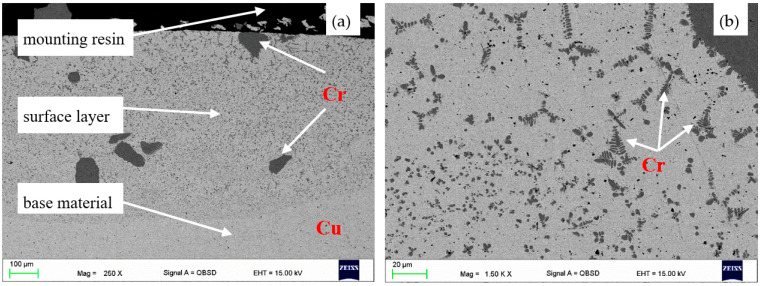
Cross-sectional structure of the coating obtained after fusing CrAl powder Cu-ETP copper, (**a**) surface layer zones, (**b**) chromium dendrites; laser power, melting rate; SEM.

The surface layer produced on the Cu-ETP surface with the help of chromium and aluminium powder can be classified as a quasi-coating. There is clear difference between the base material and the remelted surface layer marked with border boundary: in the surface layer, the fed chromium occurs in forms, such as in the bulk phase, as well as in form of very small precipitates acting as the precipitation strengthening factor. However, it is clearly separated from the ground (Figure 3a). In the quasi-structure, the chromium precipitates take the shape of fish bones (Figure 3b).

**Figure 4:**
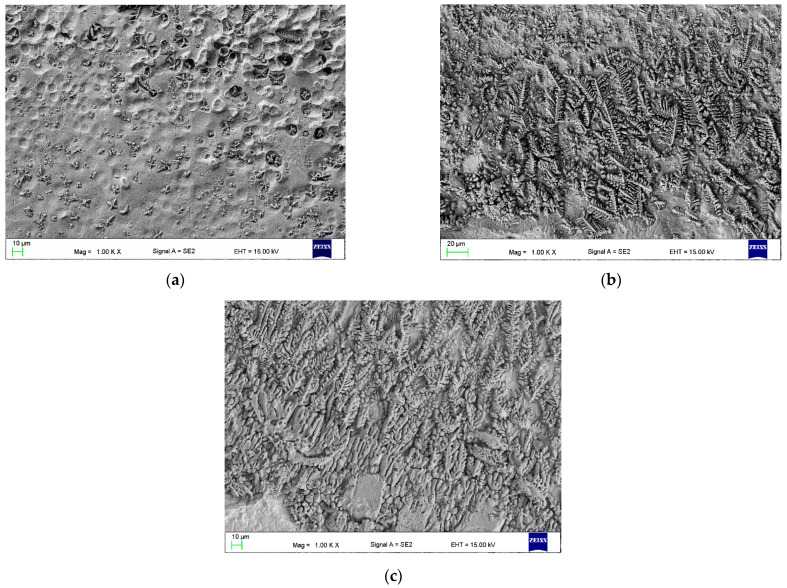
Cross section of the top layer of the Cu-ETP guide after fusion of CrAl powder: powder feed rate (**a**) 0.2 mm^3^/min; (**b**) 0.4 mm^3^/min; (**c**) 0.8 mm^3^/min; SEM.

Figure 4 shows the cross sections of the top layer after the CrAl powder has fused in. In the laser-remelted layer of chromium, the solubility of which in copper is very low, it takes the form of dendrites. Depending on the remelting parameters, the amount of dendrites in the observed space is very large, small (Figure 4a), or large (Figure 4c). The reason for the large dendrites is a relatively low cooling rate, which allows the dendrites to groove. It should be noted that samples were not cooled after laser treatment. Moreover, the presence of smaller Cr dendrites testify to a difference in the cooling rate in the remelted layer. The aluminium used for alloying does not form any large structures such as bulk precipitates or dendrites, so it will be further revealed that the aluminium occurs in form of smaller praticles not visibile in this range of magnification.

**Figure 5:**
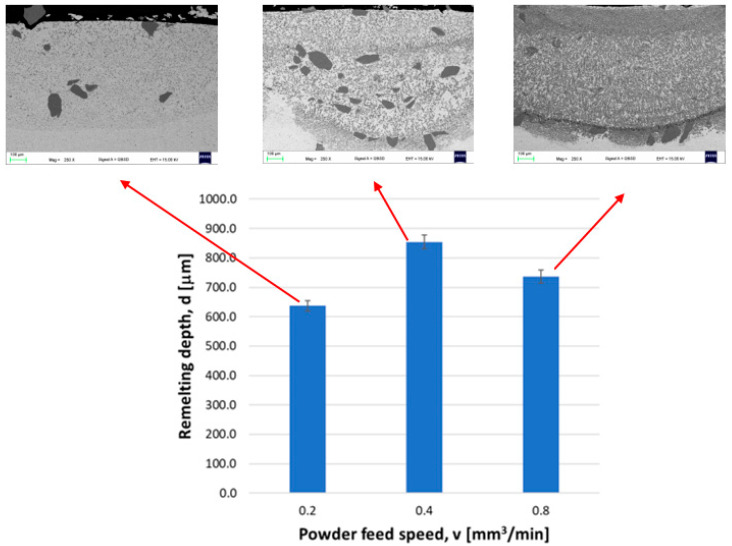
Effect of the powder feed rate on the thickness of the formed layer.

In Figure 5, the relation between the powder feed rate and the respective layer thickness is presented. What was unexpected, on the basis of analysis of other studies [9,10,13], is that the increase of the feed rate causes the increase of the layer thickness. The highest measured thickness layer value corresponds to the feed rate of 0.4 [mm^3^/min]. A reason for the increase of layer thickness could be a result of the higher values of the powder feed rate, which reached more than 0.8 [mm^3^/min]. This could be due to the laser beam energy absorption by the additional amount of powder, as the powder particles accumulate heat energy and are therefore able to remelt deeper regions of the treated surface layer.

**Figure 6:**
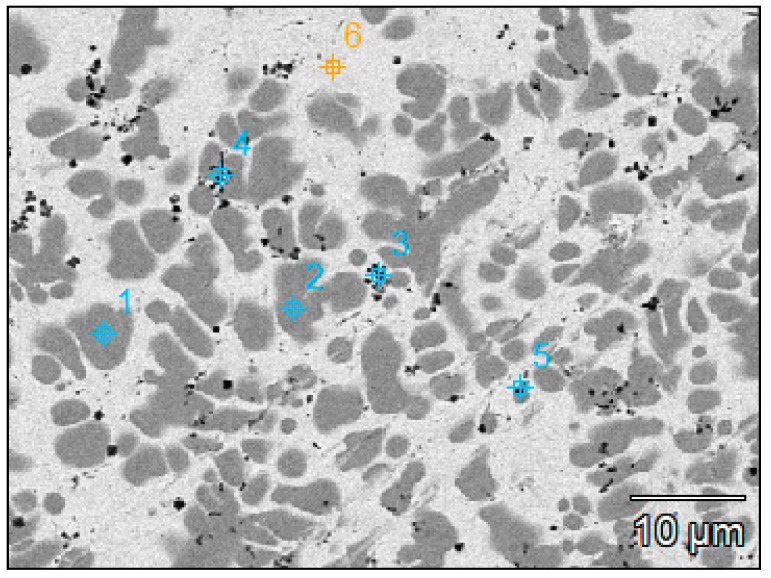
Microstructure of the cross section of the remelted surface layer after CrAl powder laser treatment; the marked points are explained in Table 1; SEM.

On the basis of the results of the X-ray qualitative and quantitative EDS microanalysis, the presence of the elements Cu as a matrix component and Al and Cr, i.e., the elements included in the fused powder, was confirmed. Moreover, it was found that in items 1 and 2, the chromium concentration was 96 wt.%. (Figure 6, Table 1) while the copper concentration was about 4%. In turn, in item 6, which characterizes the matrix, the chromium concentration was only 4.1 wt.%. while copper was 95.9 wt.%. In areas 3, 4, and 5, which in the microstructure in Figure 6 are visible as heterogeneously distributed, small, almost spherical precipitates, there is copper (25.4–86.5% by weight), chromium (11.9–65.0% by weight), and aluminium (1.6–9.6% by weight).

**Figure 7:**
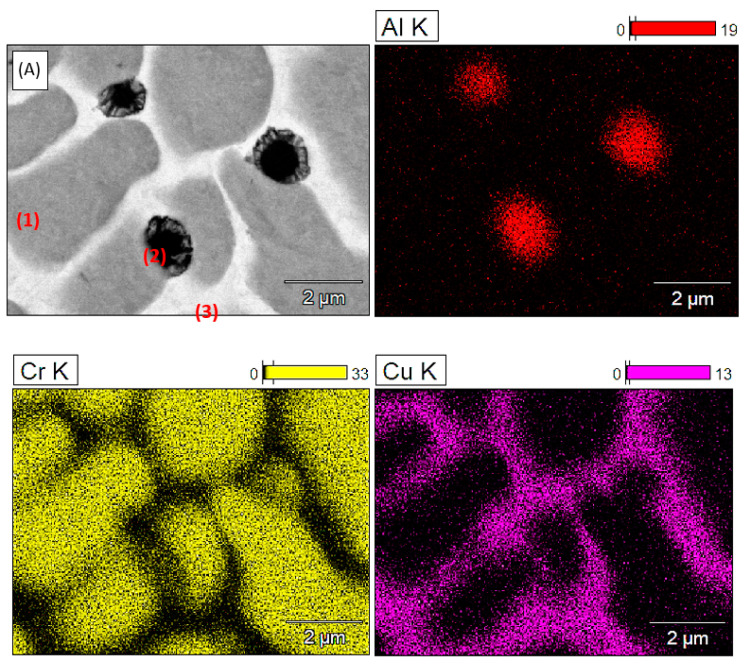
The microstructure of the surface of the top layer after fusion of the CrAl powder into the guide surface of the Cu-ETP copper section insulator; image obtained with the use of secondary electrons (A) and a map of the surface of the distribution of the elements Al, Cr, and Cu.

The distribution of alloying elements in the microstructure of the alloyed Cu-ETP + CuAl layer is shown in Figure 7. Based on the results of the X-ray qualitative and quantitative EDS microanalysis, it was found that there was a clear segregation of the alloying elements. In large precipitates, (1) chromium dominates, in smaller precipitates with a spherical shape (2) aluminium dominates, while the matrix was identified almost exclusively by copper.

**Figure 8:**
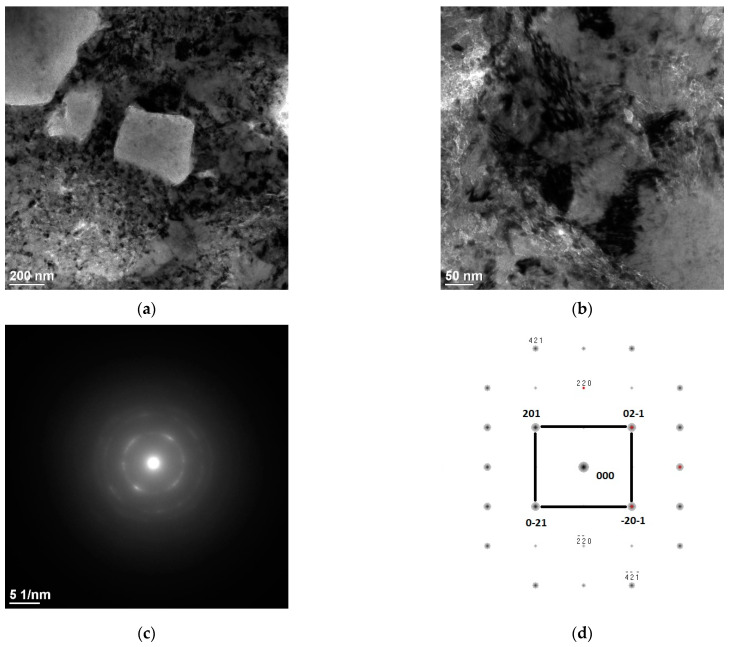
Results of the investigation of TEM microstructures, (**a**) bright field image Cr phases, (**b**) Cu matrix, (**c**) SAED pattern from the areas in (**a**), (**d**) solution of the diffraction pattern presented in (**c**).

In the investigation of the microstructure of the transmission electron microscope, as well as electron diffraction analysis, the Cr phase particles were confirmed on the SAED diffraction (Figure 8c). As the chromium phase, the cubic crystallographic phase was recognised with the lattice parameters of a = b = c values equal to 2.97 Å and the angle α = β = γ equal to 90°, as well as with the zone axis of the [1-1-2] direction. The chromium particles present in the copper matrix are 200 nm or higher, as shown in the SEM. The morphology is uniform in this range of size; however, the nature of Cr precipitation is different from that of the equilibrium diagram one. In addition, the microstructure of the matrix material reveals a relatively high level of diversity of the microstructure compounds, which comes from the high energy level delivered by the laser beam. The high amount of energy leads to obtaining a very non-homogeneous structure compared to standard heat treatment, which is desirable for the task of the treated material surface, ensuring a longer lifetime by stability of the obtained properties, because a non-homogeneous structure delivers higher mechanical properties, according to the Hall–Petch equation, as well as according to dispersion strengthening.

**Figure 9:**
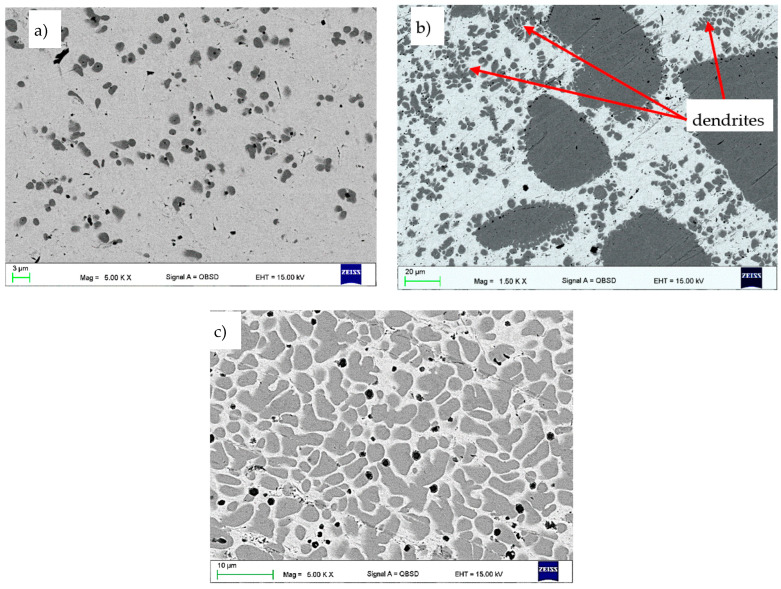
Cr particle morphology in the Cu matrix, cross section of the top layer of the Cu-ETP guide after fusion of CrAl powder: powder feed rate (**a**) 0.2 mm^3^/min; (**b**) 0.4 mm^3^/min; (**c**) 0.8 mm^3^/min; SEM.

As shown in Figure 9, chromium particles can take both an oval shape (Figure 9a,b) and a shape close to oval with rounded edges (Figure 9c). They can also take the shape of dendrites (Figure 9b). Chromium particles are arranged irregularly in the Cu matrix, which is particularly visible in Figure 9a,b, i.e., for the powder feed rate of 0.2 mm^3^/min; 0.4 mm^3^/min.

Unlike in the equilibrium ternary phase equilibrium diagram, in the studied case the cooling rate significantly exceeds the conditions close to equilibrium. For this reason, the phase composition in the laser-processed component may differ from the phase composition in the ternary equilibrium system. For this reason, the obtained results of structural and/or phase composition tests can be used—although it was not the main research intention of the research presented in the article—to create a complete Cu-Cr-Al equilibrium system.

Experimental thermodynamic data of phase equilibria are known only for the relevant binary systems. Recently, the ternary system has received renewed interest because of the ability to synthesize quasicrystals in Al-rich compositions. In particular, compositions around Cr15Cu20Al65, Cr20Cu10Al70, Cr15CuxAl85-x (x = 0–20) have been investigated extensively [14].

### 3.2. Qualitative X-ray Phase Analysis

**Figure 10:**
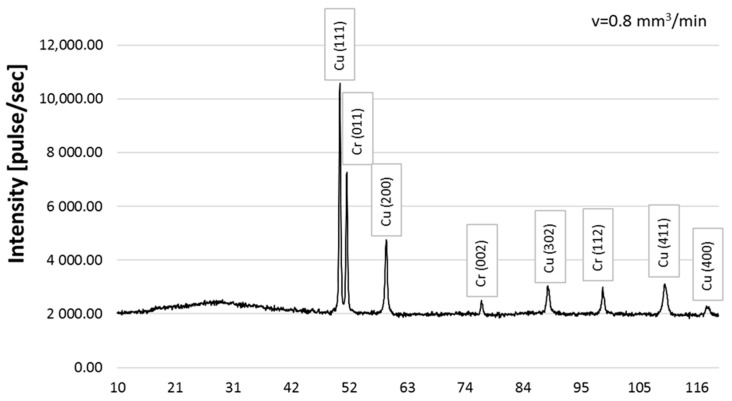
X-ray diffraction pattern for the qualitative CrAl coating on Cu-ETP copper, powder feed rate v = 0.8 mm^3^/min.

**Figure 11:**
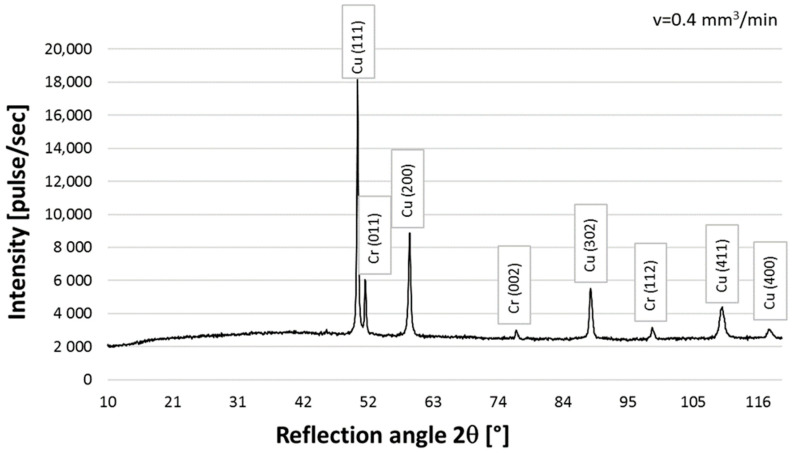
X-ray diffraction pattern for the qualitative CrAl coating on copper Cu-ETP, powder feed rate 0.4 mm^3^/min.

**Figure 12:**
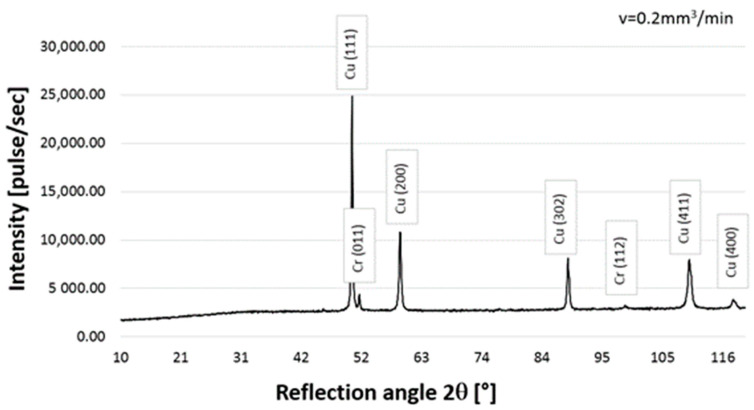
X-ray diffraction pattern for the qualitative CrAl coating on copper Cu-ETP, powder feed rate 0.2 mm^3^/min.

The following phases were found in the structure of the CrAl coating on copper Cu-ETP based on the qualitative X-ray phase analysis: copper Cu-peaks coming from planes (111), (200), (302), (411), and (400) and chromium Cr-peaks from planes (011), (002), and (112) (Figure 10). Regardless of the powder feeding speed, during laser surfacing (0.2–0.8), the diffraction patterns show peaks from the same planes, and only the intensity of the peaks differs (Figure 10, Figure 11 and Figure 12). Similar results were obtained in Ref. [15].

Moreover, on the basis of the diffractogram analysis, it was noticed that only copper and chromium were present in the coating structure. No other phases were found, which corresponds to the phase equilibrium system presented in Ref. [12].

**Figure 13:**
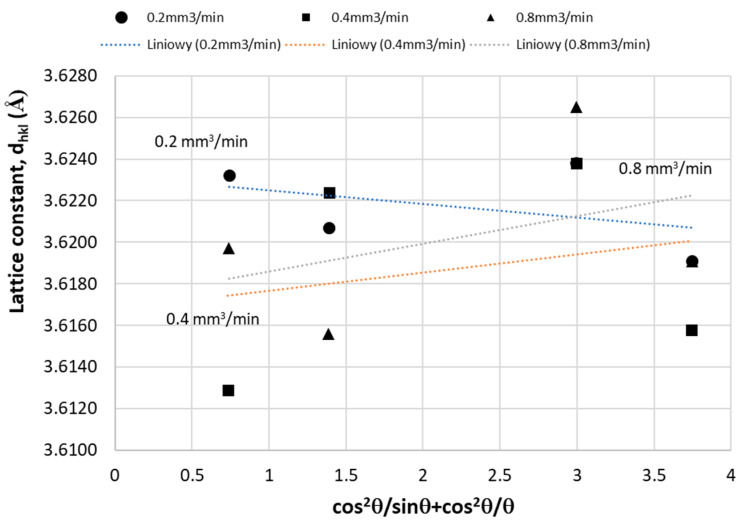
Variation of the lattice parameters of Cu (αCu) as a function of the Nelson–Riley (N-R) parameter [16]; for analysis, the four strongest XRD picks from Figure 10, Figure 11 and Figure 12 were used.

Figure 13 shows the change of the Cu_(aCu)_ matrix lattice parameter of Cu-ETP as a function of the Nelson–Riley (N-R) parameter for the respective peaks obtained from the XRD analysis of the test samples:

(1) $$\left(\frac{{cos}^{2}\theta }{sin\theta }\right)+\left(\frac{{cos}^{2}\theta }{\theta }\right)$$

The functional relationship (2) is advantageous to determine the exact parameters of the network ($a_{Cu}^{0}$) by a suitable extrapolation [16].

For the lattice parameters calculations, the stronger XRD pics were used according to the crystallographic state of the art (Figure 13). 

### 3.3. Hardness of the Surface Layers

**Figure 14:**
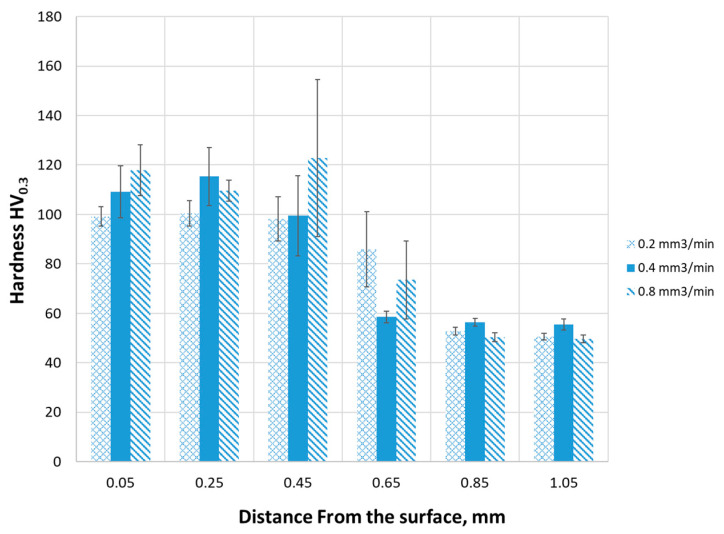
Change in hardness in distance from the coating surface.

The hardness of the Cu-ETP guide before remelting was 60 HV. In the sample remelted with a laser with a powder feed speed of 0.2 mm^3^/min to a depth of 0.4 mm, an almost two-fold increase in hardness can be seen. On the other hand, at a depth of more than 0.6 mm, the hardness of the coating takes the value of the base material (Figure 14). This suggests that the coating layer has a maximum thickness of approximately 600 µm. The maximum mean hardness value was obtained for the depth of 0.25 mm, which was 115 HV_0.3_. The maximum measurement value for this depth was 142 HV_0.3_ (1 of 8 measurements) with a standard deviation of 11.73, which is 10% of the mean value.

On the other hand, in the sample remelted with a laser with a powder feed rate of 0.4 mm^3^/min, the highest hardness was measured for a distance from the surface of 0.05 mm; 0.25 mm, and 0.45 mm, which were, respectively, 99 (standard deviation 3.8), 100 (standard deviation 5.2), and 98 HV_0.3_ (standard deviation 8.9). The highest values of single measurements were recorded for the distance from the surface of 0.25 mm and 0.45 mm, respectively, 111 and 110 HV_0.3_. Hardness measurements at a greater distance from the surface showed a decrease in hardness to the value of the Cu-ETP guide material.

However, in the sample remelted with a laser with a powder feed rate of 0.8 mm^3^/min, the highest hardness was observed among all the samples, which for a distance from the surface of 0.45 mm was 123 HV_0.3_. In comparison to the hardness of the base material of the Cu-ETP guide, it is a two-fold increase in hardness. The hardness of this sample, measured at a distance from the surface of 0.05, 0.25, and 0.45 mm is 118, 110, and 123 HV_0.3_, respectively. For the value of 118 HV_0.3_, the standard deviation is 10 HV_0.3_; for a hardness of 100 HV_0.3_–4.3 HV_0.3_ and for the highest measured value, 123 HV_0.3_ is as much as 31 HV_0.3_, which is almost 27% of the average value (Figure 14). It should be assumed that at this depth there is a large amount of precipitates of chromium, which is responsible for the increase in hardness; however, the distribution of these particles is definitely uneven and there may be local clusters of chromium dendrites, as evidenced by a very high value of the standard deviation (27% of the average value).

When comparing the highest hardness values for the three tested samples, it was observed that they were in the range of 98–123 HV_0.3_, regardless of the CrAl powder feed rate used (Figure 14). For all samples, the highest hardness values were recorded for the distance from the surface in the range of 0.05–0.45 mm.

For all samples, the hardness at a distance from the surface of 0.85 and 10.5 mm is 50–56 HV_0.3_, which is below the hardness of the base material. This slight reduction in hardness is most likely caused by the alloying provided in the process., where the original microstructure of the copper base material was destroyed by the laser beam energy, and a growth of the grain size took place. As we already know, larger grains are the reason for the lower hardness, according to the Hall–Petch equation [3]:

(2) $$R_{e}=R_{0}+\frac{k}{\sqrt{d}}$$

where:

*R_e_*—Yield strength of the material

*R*_0_—Yield strength of a single crystal (stress of lattice wear)

*k*—Material constant;

*d*—Average grain size

On the other side, a higher hardness value, obtained for the smaller distance from the surface reaching up to 0.45 mmm from the surface, is caused by a relatively high energy density of the molten material, and therefore a higher gradient of the cooling rate, causing the nucleation process to be interrupted, and more small grains could therefore come into existence. More small grains mean a higher tension between the boundaries, and as a result, a higher hardness compared to material heat treated using standard procedures, e.g., for quenching or ageing after solution heat treatment.

A second reason for the strengthening of the surface layer up to 0.45 mm after the laser alloying was the possible occurrence of solution strengthening involving the addition of chromium powder. However, some additional investigations could be provided here, especially using a transmission electron microscope for detecting the possible strengthening phases, as even small Cr particles could be present in the remelted Cu matrix.

### 3.4. Wear Resistance

**Figure 15:**
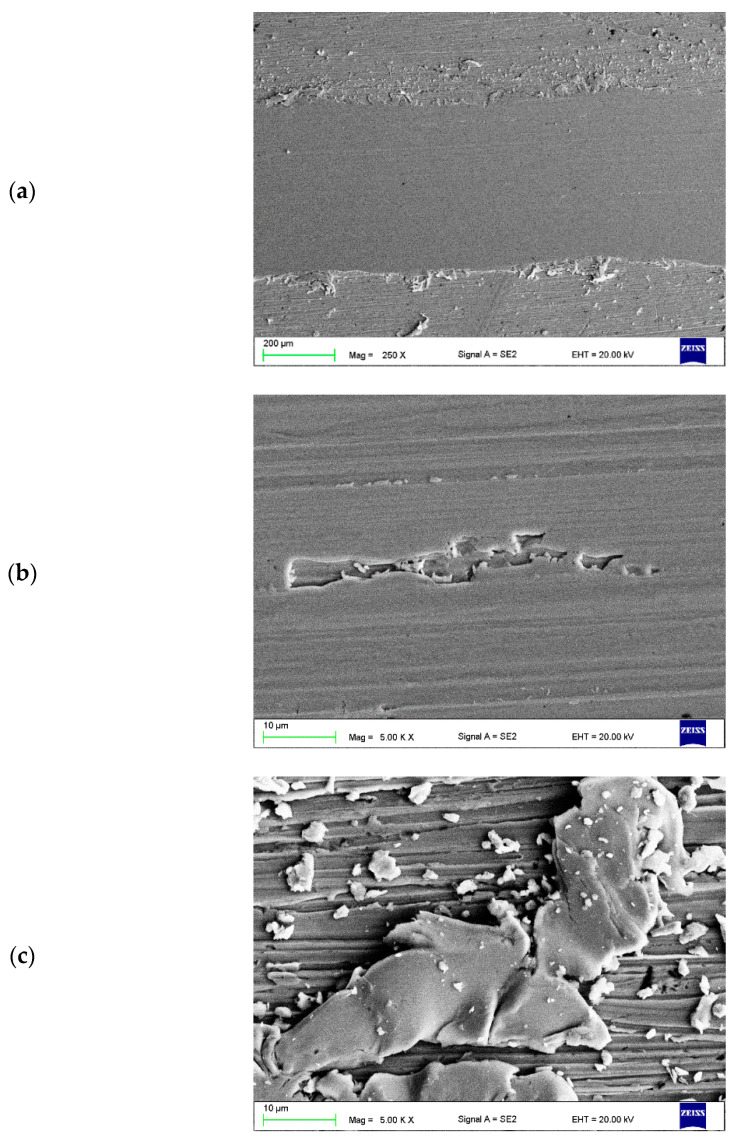
Wear track (**a**,**b**) of the tested Cu-ETP substrate and (**c**) wear debris.

In the next stage of the investigation, an analysis of the abrasion trace made by the countersample of ZrO_2_ was performed. In the Cu-ETP base material, the edges of the wear track are parallel and straight. The wear track surface is smooth with numerous visible grooves parallel to the sliding direction, with abrasion via ploughing (Figure 15a,b). Furthermore, some traces of delamination were observed (Figure 15b). Friction products (wear debris) can only be observed outside the wear track. The wear debris consisted of tiny particles about 1 µm in size and large flakes with visible signs of plastic deformation (Figure 15c).

**Figure 16:**
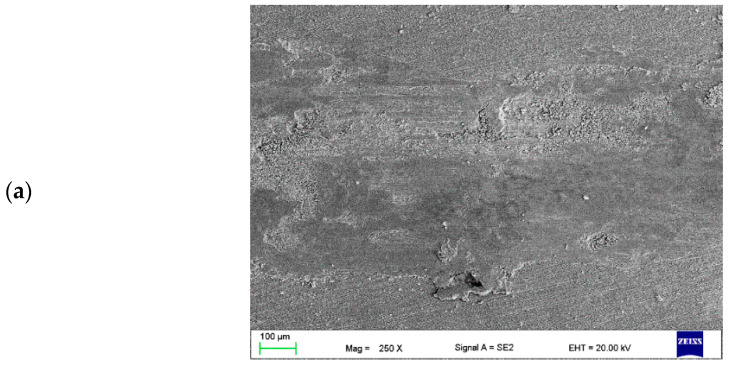

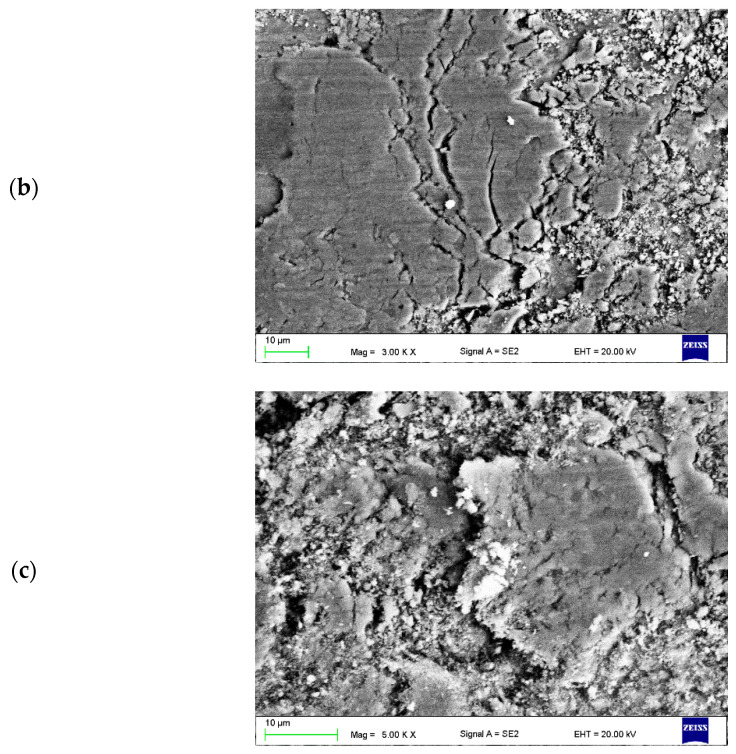
Wear track (**a**,**b**) of the tested CrAl v = 0.8 mm^3^/min layer and (**c**) wear debris.

**Figure 17:**
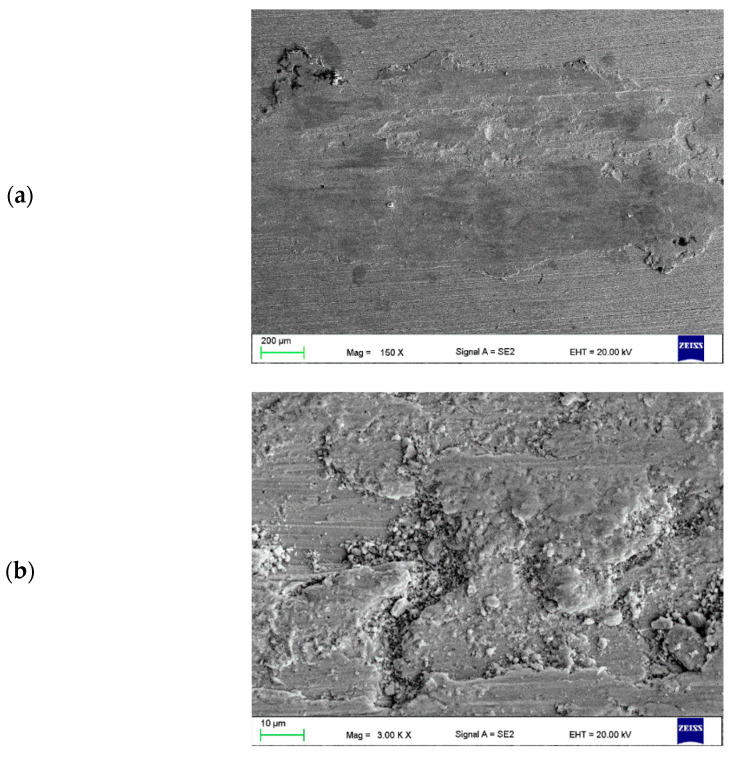

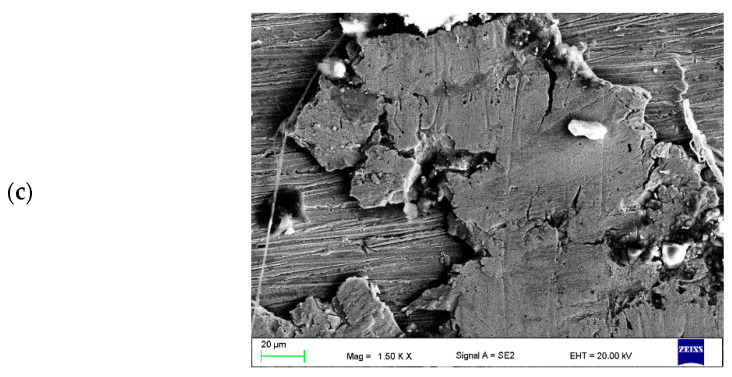
Wear track (**a**,**b**) of the tested CrAl v = 0.4 mm^3^/min layer and (**c**) wear debris.

**Figure 18:**
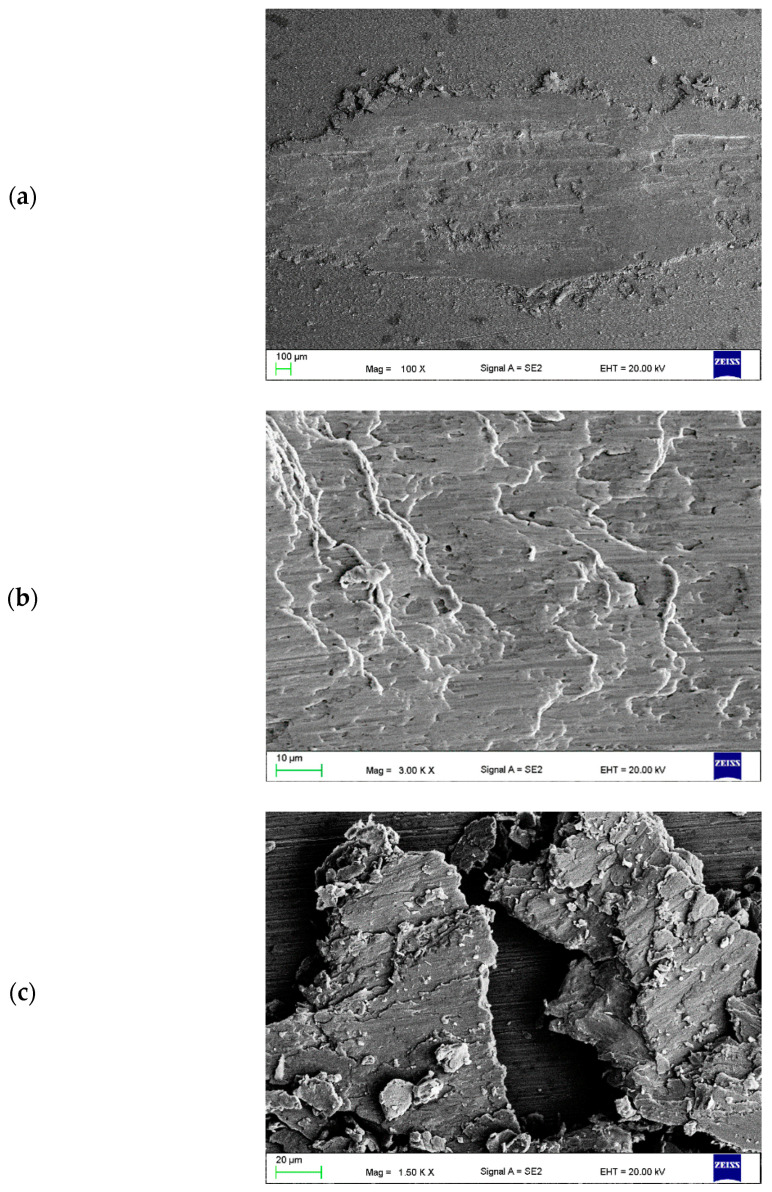
Wear track (**a**,**b**) of the tested CrAl v = 0.2 mm^3^/min layer and (**c**) wear debris.

In the case of all CrAl layers, the edges of the wear track are uneven (Figure 16a, Figure 17a and Figure 18a). Furthermore, numerous grooves parallel to the sliding direction were observed on the wear surface. Numerous traces of plastic deformation were revealed in the case of the layer with the highest volume of powder introduced (Figure 16b). Wear debris consisted of individual flakes up to 15 µm in size and fine dust for this layer. In addition, the most significant oxidation was visible (very bright areas). Numerous signs of delamination and fatigue wear were observed on the wear track surface of all layers (Figure 16b, Figure 17b and Figure 18b). Wear debris after the tribological test of the layers with the average and lowest amount of powder introduced consisted mainly of large metal pieces detached from the substrate (Figure 17c and Figure 18c).

**Figure 19:**
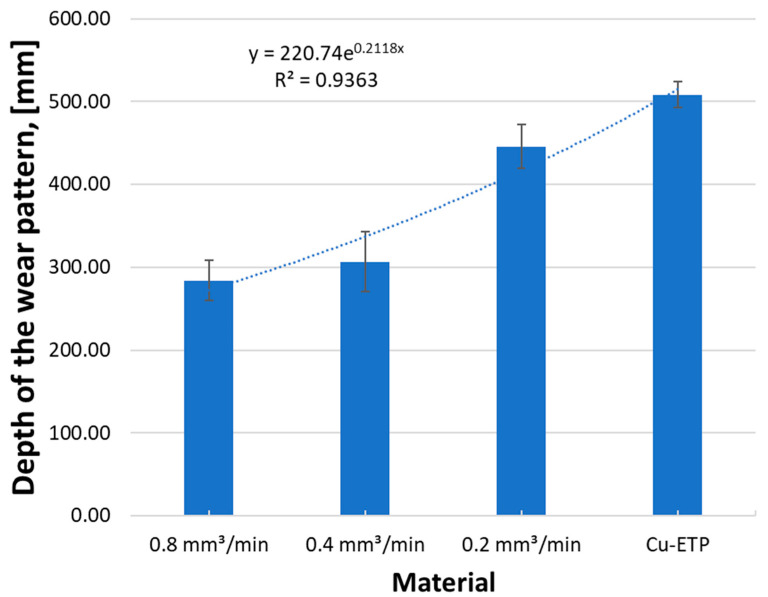
Effect of the CrAl powder feed rate during laser remelting on the depth of the wear trace.

The influence of the powder feed rate during laser remelting on the depth of the wear trace is shown in Figure 19. It was found that the CrAl coating produced by laser remelting of the surface layer provides greater wear resistance. This is visible primarily at the smaller depth of the abrasion band for the Cu-ETP + CrAl layer for all manufacturing parameters. 

**Figure 20:**
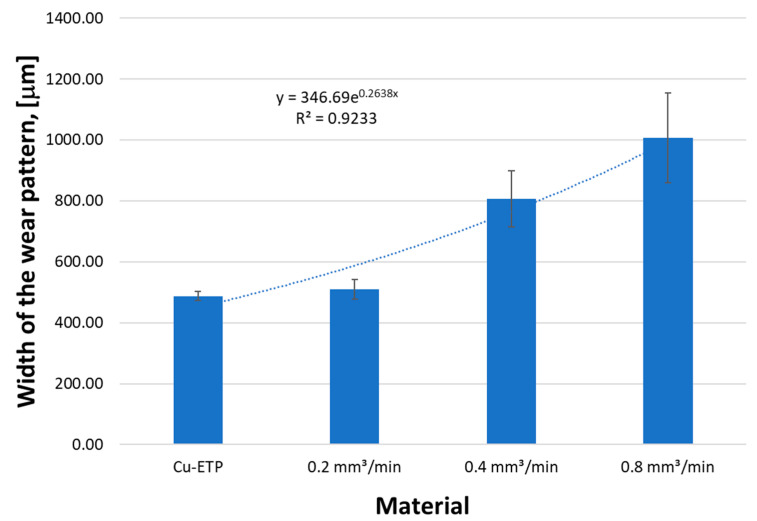
Effect of the CrAl powder feed rate during laser remelting on the width of the wear trace.

The smallest wear trace width was found in the native material of the Cu-ETP guide s = 487 mm with the standard deviation σ = 14.9, which is 2% of the mean width value (Figure 20). Among the modified surfaces, the smallest wear track was observed for the CrAl coating v = 0.2 mm^3^/min and the greatest abrasion track width of the wear track v = 0.8 mm^3^/min.

**Figure 21:**
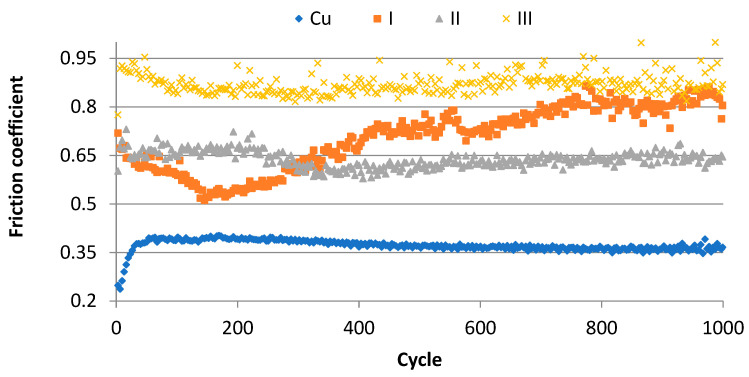
Comparison of the friction coefficient of the modified Cu-ETP copper surface; Cu-Cu-ETP substrate; I-CrAl coating v = 0.2 mm^3^/min; II-CrAl coating v = 0.4 mm^3^/min; III-CrAl coating v = 0.8 mm^3^/min.

In the next stage of the investigation, the coefficient of friction of copper Cu-ETP and melted layers of Cu-ETP + CrAl were measured. On the basis of the results obtained, it was found that the lowest coefficient of friction is characteristic of Cu-ETP copper, which at the beginning was slightly more than 0.36, but after about 500 cycles, it stabilised at a level of 0.36 (Figure 21). In turn, the Cu-ETP + CrAl coating made at the powder feed rate v = 0.2 mm^3^/min at the beginning of the friction coefficient test decreased to a value of about 0.5, and then increased until it reached the value of 0.8 after 800 cycles. The next layer of Cu-ETP + CrAl v = 0.4 mm^3^/min is characterized by a relatively constant value of the friction coefficient, which was approximately 0.65 during the entire test. The highest friction coefficient is characteristic for the Cu-ETP + CrAl v = 0.8 mm^3^/min coating, which has a friction coefficient of approximately 0.85.

## 4. Discussion

In alloyed copper, the addition of chromium increases the tensile strength and hardness [17,18]. During the heat treatment of Cu alloys with the addition of chromium, highly dispersed Cr particles are released from the supersaturated solid solution α (Cu). This results in an increase in strength and a decrease in plasticity [18].

The laser alloying of the surface layer of the working part of the guide consisted of introducing alloying elements into the treated parent material by hydrodynamic mixing of both materials. A necessary condition for remelting is that one of the materials is liquefied. The laser beam causes the material to melt and form a puddle. As a result of gravitational and convective movements as well as the pressure of the laser beam, the materials mix [10]. The mixing of the alloyed material can be of different kinds; sometimes there are particles detected only on the bottom of the metal lake, sometimes only in the upper region. Of course, the best option would be to have a relatively homogeneous mixture in the entire region of the remelted area of the surface layer. Regarding the external effects of the remelting, there could also be differences between the materials and processing parameters. In this case, there is, for example, an outflow at the edge of the lake. This process depends mainly on the temperature gradient, the concentration gradient of diffusing elements, and the diffusion time [19,20].

In CuCr alloys with a chromium content below 2%, chromium crystallises in the form of highly dispersed particles in interdendritic spaces. Due to the heterogeneous distribution of chromium particles (Figure 3b), it significantly affects the electrical conductivity of the layers produced. This structure defect can be eliminated by applying heat treatment, supersaturation, and aging [21].

The depth of diffusion saturation with the alloyed element usually does not exceed 10 μm, which is less often when the diffusion zone reaches the depth of 200–300 μm. This is because the laser beam does not allow for diffusion, causing the formation of large temperature gradients in a very short time [13]. However, in this experiment, the calculated depth of fusion of the working surface of the Cu-ETP guide with CrAl powder for the parameters of the first variant is 637 μm, for the second variant it is 854 μm, and for the third variant it is 736 μm (Figure 5).

This condition can be explained by the very low solubility of chromium in copper, as well as the fact that chromium does not form chemical compounds with copper in commercial alloys. Chromium solubility increases with temperature and reaches its maximum value of 0.73% by weight at a temperature of 1076.6 °C. However, at ambient temperature, it is only 0.03% [12]. As the concentration of chromium in copper increases, the resistivity of the alloy increases and with each addition of 1 wt.%, Cr increases to 4.9 μΩ*cm [22]. The limit of chromium in copper is determined by the following relationship [23]:

(3) $$C_{Cr}=1745exp\left(\frac{-15450}{RT}\right)$$

where: 

*C_Cr_*—chromium concentration in wt.%; 

*R*—universal gas constant; 

*T*—temperature

Interpretation of the structure obtained on the basis of the collected research results encounters great difficulties related to the fact that the description of the structure of CuCr alloys and the mechanisms of precipitation of chromium particles in the Cu matrix refers to the CuCr alloy copper with a relatively low chromium content (usually up to about 2%) and the related classic heat treatment: solution with subsequent aging [24] or thermomechanical treatments [25]. Moreover, some scientific works describe the structure and properties of CuCr alloys obtained by powder metallurgy [26] or as a result of selective laser melting [27] or treatment of CuCr25 and CuCr50 alloys by a High Current Pulsed Electron Beam [28]. The coating obtained in this study is an experimental method of forming only the surface layer of chromium on Cu-ETP copper with a fibre laser beam.

As in other composites, the addition of a component representing completely different properties, as in the case of Cu-ETP copper, the addition of chromium strengthens the matrix, which increases the mechanical and tribological properties, etc. [29]. 

The results obtained for the friction coefficient were compared with Ref. [30], where the friction coefficient between pure copper (99.9%) and graphite (DE9000) was obtained in the range of 0.2–0.5 depending on the applied force. However, in Ref. [31], the friction coefficient between pure copper (99.99%) and graphite (C 84.5%; O 10.2%, the rest of S) was obtained at the level of 0.12–0.13 depending on the test speed (30–180 km/h). The latter values are consistent with the results obtained in Ref. [32] for the copper Cu-ETP pair and for various carbon materials for which the friction coefficient is set at 0.116–0.147. As you can see, all these coefficients are lower than those obtained in this study. This difference may result from the nature of the coating as well as the ZrO_2_ counterpart used. Another advantage presented in this work is the practical nature of the investigations started and provided by Dutta [19], namely the application of laser remelting and alloying in the means of railway transport for the surface modification of traction section insulators. For this task the fibre laser was used instead of the old-type CO_2_ laser, with innovatory features such as a more precisely worked laser beam control. Another advantage of the fibre laser used here is a relatively higher power density caused by the laser in the upper part of the treated surface layer, which leads to an even higher maximal temperature value achieved during the remelting process, compared to the CO_2_ laser. This fact makes it possible to obtain a higher solidification rate of the molten material, which is the reason for refinement of the microstructure, and therefore the lower grain size. As we know, a smaller grain size in the microstructure is the reason of higher hardness, because of the internal stress in the regions between the grain boundaries. In this work, this result was observed during the analysis of the hardness measurement, which is presented in Figure 14, where the grainsize refinement reaches a depth of ca. 0.45 mm into the treated surface. Reassumed, after the Dutta investigations we were able to obtain a more hard surface layer due to a more intensive cooling speed of the molten and/or alloyed material of the surface.

## 5. Conclusions

As part of the experiment, CrAl coatings were made on Cu-ETP copper in the laser alloying process using a fibre laser. Based on the test results of the obtained coatings, it was found that:

- The layers thickness of the obtained layer when smelting copper Cu-ETP with CrAl powder varies between 600 and 850 µm; the speed of powder feeding affects the thickness of the layer produced. The layer has the smallest thickness for v = 0.2 mm^3^/min and the highest for v = 0.4 mm^3^/min;
- As a result of the low solubility of chromium in copper, it was found that in the microstructure of the layer chromium dominates in large precipitates, whereas in the matrix it was identified almost exclusively with copper;
- The Cu-ETP + CrAl layers are characterised by a higher coefficient of friction than the Cu-ETP starting material, which for the layer, the CrAl coating v = 0.08 mm^3^/min, increased almost three times;
- For all the coatings produced, the highest hardness was recorded for the distance from the surface in the range of 0.05–0.45 mm, with the maximum hardness of the coatings being 98–123 HV_0.3_ compared to the base material Cu-ETP 60 HV_0.3_;
- On the basis of the analysis of XRD patterns, only two phases were found: copper and chromium.
- After the laser remelting/alloying of the Cr powder, the Cr precipitations are very well distributed in the Cu matrix, reaching a size of 200 nm, which makes the laser beam surface more stable according to the Hall–Petch equation, ensuring higher hardness and wear resistance compared to standard heat treated element. This should significantly extend the lifetime of the section insulator guides working under real conditions.

## Figures and Tables

**Table 1:** Results of the quantitative EDS microanalysis of the chemical composition of the surface layer after fusion of CrAl powder into the surface of the guide rail and the sectional element made of Cu-ETP copper.

	Al		Cr		Cu	
	Wt, %	At, %	Wt, %	At, %	Wt, %	At, %
Base(11)_pt1	–	–	96.2	96.9	3.8	3.1
Base(11)_pt2	–	–	95.9	96.6	4.1	3.4
Base(11)_pt3	5.3	10.4	59.2	60.1	35.5	29.5
Base(11)_pt4	9.6	17.7	65.0	62.3	25.4	20.0
Base(11)_pt5	1.6	3.7	11.9	13.8	86.5	82.5
Base(11)_pt6	–	–	4.1	5.0	95.9	95.0

## Data Availability

Not applicable.
